# Directional Motion of Coalesced Viscous Droplets on Fibers with Wettability Gradients

**DOI:** 10.1021/acsomega.5c12758

**Published:** 2026-02-17

**Authors:** Zeming Fu, Huagen Wu, Yanling Xiong, Paolo Tronville

**Affiliations:** † School of Energy and Power Engineering, 12480 Xi’an Jiaotong University, Xi’an 710049, China; ‡ Department of Energy, 19032 Politecnico di Torino, Turin 10129, Italy

## Abstract

The coalescence and directional migration dynamics of oil droplets on wettability gradient fibers were investigated based on the volume of fluid (VOF) method combined with an improved dynamic contact angle model. We clarified the effects of initial configuration, wettability gradient, and liquid viscosity on droplet morphology, migration velocity and viscous dissipation. The results indicate that after coalescence, droplets migrate directionally along the wettability gradient, and a larger gradient leads to a higher migration velocity. Low viscosity droplets exhibit noticeable oscillations during acceleration, while high viscosity droplets move more smoothly due to increased energy dissipation. As viscosity increases from 0.024 Pa·s to 0.093 Pa·s, normal strain dissipation dominates the total viscous dissipation, accounting for about 63% at the peak stage, corresponding to liquid bridge formation and strong droplet deformation. The average sensitivity of maximum velocity to viscosity is approximately 7.5%, with stronger competition between driving and resistive forces in the low viscosity regime and a transition to viscosity dominated behavior at higher viscosities. A stronger wettability-driven force slightly weakens the suppressive effect of viscous resistance and increases kinetic energy conversion efficiency. These findings provide insight into the migration dynamics of microdroplets driven by wettability gradients.

## Graphical abstract

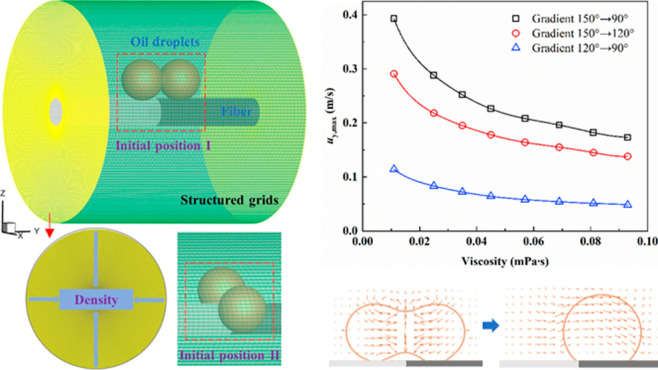

## 1 Introduction

Fibrous coalescence filters are widely employed in industrial processes such as oil-lubricated compressors, chemical gas purification, gas turbine intakes, and building air pollution control,^1,2^ with the primary function of efficiently removing liquid aerosols from gas streams. After being captured by fibers, aerosol droplets migrate along the fiber surface or undergo coalescence through mutual collisions, eventually draining as large droplets or liquid films.^3^ The wettability of the medium is a critical determinant of separation performance, as it governs the interactions between droplets and fibers, thereby dictating droplet migration and distribution within the fiber layer and ultimately influencing the overall removal efficiency of the filter material.^4,5^ The interfacial dynamics of the gas–liquid phases and the local microscale flow behavior govern the fundamental separation mechanisms of such fibrous structures, imparting inherently microfluidic characteristics to these processes. The mechanisms that regulate droplet behavior are also closely aligned with the wettability-based control strategies commonly encountered in micro and nanofluidic systems.^6^

In recent years, researchers have investigated the influence of fundamental filter parameters and operating conditions on separation performance.^7,8^ The wettability plays a pivotal role in oil-mist filtration by fibrous materials. A common approach to modulating wettability is surface modification of the filter medium.^9^ Kampa et al.^10^ proposed the Jump & Channel theory, which interprets pressure drop variations between oleophilic and oleophobic materials at different stages of oil-mist separation based on mechanistic insights from observed pressure drop curves. Mullins et al.^11^ demonstrated that filters composed of mixed wettability media exhibit distinct behavior, with oleophobic materials reaching a lower steady state saturation than oleophilic ones. Moreover, placing an oleophobic layer as the final stage yielded superior overall performance. Penner et al.^12^ examined filters composed of various media combinations, including two types of oleophilic and two types of oleophobic materials. They found that using a coarse-pore medium upstream markedly reduced pressure drop while slightly enhancing separation efficiency. Wei et al.^13^ fabricated filter media with a wettability gradient along the thickness direction, enabling directional droplet migration. Their study showed that surface energy contrast plays a unique role in unidirectional liquid transport, offering a new strategy for accelerated drainage. Mandic et al.^7^ reported that fiber wettability is critical to both liquid retention and drainage processes, with oleophobic fibers facilitating the detachment and removal of “clamshell” coalesced droplets from the surface. Chang et al.^14^ investigated asymmetrically wetted filter media prepared by surface modification, demonstrating that adjusting the treated area ratio, spray coating duration, and pattern design can substantially improve integrated filtration performance under specific conditions. Collectively, these studies, which range from mechanistic modeling and structural optimization to functional gradient design, highlight the crucial role of wettability regulation in enhancing coalescence filtration. Filters with different wettabilities exhibit significant differences in pressure drop behavior, droplet attachment morphology, and the underlying separation mechanisms.^15^

The operation of oil-mist filters relies on the synergistic action of the coalescence layer and the drainage layer.^16^ Oleophobic media have attracted wide attention due to their ability to reduce equilibrium saturation and pressure drop, thereby improving drainage performance. The drainage layer is typically composed of hydrophobic coarse fibers, which accelerate liquid removal and lower the overall pressure drop.^17^ The use of heterogeneous wettability in oil mist filters represents an advanced material fabrication technique.^18^ Introducing hydrophobic fibers with a wettability gradient into the drainage layer is expected to enhance liquid transport further, improve saturation characteristics, and increase separation efficiency. Previous studies have shown that droplets on hydrophobic surfaces may undergo spontaneous jumping.^19^ Farokhirad et al.^20^ noted that this phenomenon generally occurs when the Ohnesorge number (Oh) is below 0.3. However, due to the relatively high viscosity of lubricating oils, coalesced oil droplets rarely exhibit jumping behavior. Consequently, the coalescence dynamics under wettability-gradient conditions, particularly the influence of droplet viscosity on coalescence and migration, remain to be further elucidated. Although wettability-gradient-driven droplet transport has received considerable attention in recent years, existing studies have predominantly focused on planar substrates with uniform geometric features. In contrast, investigations into the wetting response, gradient-driven dynamics, and migration behavior of viscous droplets on curved fibrous surfaces remain remarkably limited.^21–23^ In fibrous coalescence filtration and related multiphase flow studies, a single fiber is commonly regarded as the basic physical unit for investigating interactions between droplets and fibers. Previous studies have demonstrated that the complex droplet behavior observed in multifiber filter media largely originates from local interactions at the single fiber scale, and that the macroscopic characteristics can be viewed as a superposition of multiple single fiber processes. Therefore, single fiber models are widely employed to elucidate fundamental physical mechanisms and to provide a theoretical basis for understanding droplet migration and energy dissipation in multifiber systems.

In this study, an improved dynamic contact angle model was combined with the VOF numerical method to comparatively analyze the coalescence and migration dynamics of droplets initiated from two typical positions. We gave particular attention to the evolution of droplet morphology and velocity, and viscous effects. The results elucidate the kinetic mechanisms governing droplet migration on fibers with wettability gradients and provide a theoretical basis for the optimized design of oil-mist filters.

## 2 Numerical Method

### 2.1 Numerical Model

The interaction of droplets with a single fiber involves a dynamic two-phase flow of gas and liquid. To accurately capture the gas–liquid interface, this study employed the VOF model, in which the interface is tracked through the phase volume fraction. Based on the VOF method, we carried out numerical simulations to investigate the interaction between oil droplets and fibers, effectively characterizing droplet deformation and migration on fiber surfaces. In the calculations, both the gas and liquid phases were treated as incompressible fluids, with constant density during the flow process. The corresponding mathematical model consists of the continuity equation and the momentum conservation equation^24^

1 ∇·u=0

2 $$\rho \left(\frac{\partial u}{\partial t}+u\cdot \nabla u\right)=-\nabla p+\nabla \cdot \left(2\mu D\right)+\sigma \kappa \delta_{s}n$$

here, ρ, μ, and *p* denote the fluid density, dynamic viscosity, and pressure, respectively, while u represents the velocity vector. *n*, δ_s_, κ, and σ correspond to the interface normal vector, Dirac delta function, interface curvature, and surface tension coefficient, respectively, and *D* is the deformation tensor. In the VOF method, the transport equation describes the distribution of the liquid volume fraction α. When α = 1, the liquid occupies the computational cell fully. When α = 0, the gas occupies fully. When 0 < α < 1, the cell contains both gas and liquid phases. The surface tension source term in the momentum equation is evaluated using the continuum surface force (CSF) model.^25^

3 $$\frac{\partial }{\partial t}\left(\alpha \rho \right)+\nabla \cdot \left(\alpha \rho u\right)=0$$

In computational cells containing both gas and liquid phases, the expressions for density and dynamic viscosity are

4 $$\rho =\alpha \rho_{l}+\left(1-\alpha \right)\rho_{g}$$

5 $$\mu =\alpha \mu_{l}+\left(1-\alpha \right)\mu_{g}$$

During the droplet coalescence process, droplets undergo spreading and retraction stages, accompanied by dynamic variations in the contact angle. A static contact angle alone cannot adequately characterize the actual interaction between droplets and fibers.^26^ Therefore, in this study, we applied the Kistler dynamic contact angle model during the spreading stage, as it can achieve high consistency with experimental observations in predicting droplet spreading behavior.^27^ The model introduces the capillary number Ca = μu_cl_/σ to capture how the contact angle varies with the contact line velocity u_cl_. However, the Kistler dynamic contact angle model shows limitations during the droplet retraction stage.^28^

In contrast, the Nichita model provides predictions that better reflect actual behavior in this regime.^29^ When the droplet reaches its maximum spreading state, the mechanism governing contact angle variation becomes more complex, and existing theoretical models are unable to describe it accurately.^2^ For this stage, the present study assumes a linear variation of the contact angle to approximate its gradual recovery, thereby capturing the influence of contact angle hysteresis. Accordingly, we can summarize the applied dynamic contact angle model as follows

6 $$\theta_{D}=\{\begin{array}{l} \theta_{A}=f_{H}\left(Ca+f_{H}^{-1}\left(\theta_{eq}\right)\right),ang_{cl}>0,,u_{cl}>\lambda \\ \theta_{M}=\theta_{prev}-\xi \left(x\right)\left(\theta_{prev}-\theta_{r}\right),u_{cl}\leq \lambda \\ \theta_{R}={\left(\theta_{r}-72\;Ca\right)}^{1/3},ang_{cl}<0,,u_{cl}>\lambda \end{array}$$

where θ_*A*_, θ_*M*_ and θ_*R*_ denote the contact angles corresponding to the spreading stage, the peak spreading stage, and the retraction stage, respectively. The Hoffman function is

7 $$f_{H}\left(x\right)=\cos^{-1}{(1-2\tanh(5.16\left(\frac{x}{1+1.31x^{0.99}}\right)}^{0.706}))$$

The equilibrium contact angle is denoted as θ_eq_, while θ_*r*_ refers to the receding angle, and θ_prev_ corresponds to the contact angle at the preceding time step. The term ang_cl_ indicates the cosine of the angle formed between the velocity of the contact line and the interface normal, serving to identify whether the droplet undergoes spreading or retraction. The parameter λ, whose value is 0.0029, accounts for the influence of solid surface characteristics on droplet wetting behavior. ξ­(*x*) denotes a random variable ranging from 0 to 1, representing local variations in surface properties and thereby improving the precision in capturing the complex dynamics of droplet wetting.

### 2.2 Computational Domain and Mesh

In the geometric configuration, the computational domain is defined as the space between two coaxial cylinders, as illustrated in Figure 1. This setup facilitates the generation of high-quality structured meshes. An O-type meshing scheme was employed to ensure uniform distribution and good orthogonality around the fiber. The inner cylinder represents the fiber, with a diameter of 40 μm. The external cylinder has both a diameter and a height of 320 μm, providing sufficient space to simulate the droplet motion around the fiber.

**Figure 1:**
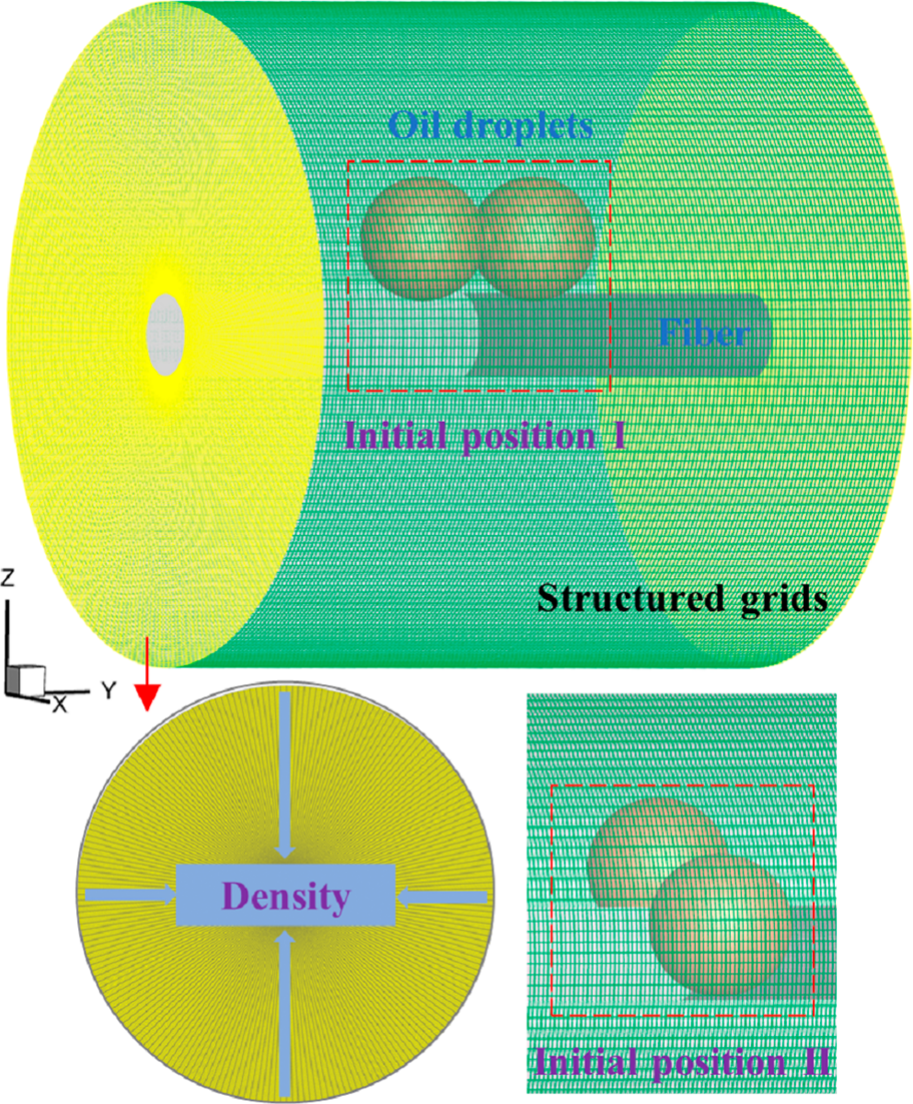
Schematic of the computational domain and structured mesh.

The initial droplet configuration considers two typical cases. In the first case, droplets are placed side by side along the fiber, with their centers aligned parallel to the fiber axis. In the second case, droplets are positioned on opposite sides of the fiber, with their centers aligned perpendicular to the fiber axis. We implement the wettability gradient by assigning different contact angles to different regions of the fiber surface. A larger contact angle is specified on the left side and a smaller one on the right side, thereby establishing a left-to-right wettability gradient.

This approach can be regarded as a numerical discretization of a physically continuous wettability gradient, with the objective of representing the monotonic variation of surface wettability along the fiber and the associated surface energy differences between regions. The physical origin of wettability-gradient-driven droplet migration lies in the overall surface energy imbalance along the three-phase contact line after coalescence, rather than being confined to local contact angle discontinuities. When the droplet spans multiple wettability regions, the resulting driving force can be equivalently interpreted as an effective wettability gradient distributed along the fiber. Therefore, the present discretized implementation does not introduce artificial spatial localization of the driving force and is capable of capturing the dominant effects of a continuous wettability gradient on postcoalescence droplet migration behavior. It should be noted that, within the continuum framework adopted in this study, the fiber surface is assumed to exhibit effective and uniform wettability within each region, and microscopic effects such as surface roughness or chemical heterogeneity are not explicitly resolved. Such effects are typically incorporated in macroscopic simulations through effective contact angle parameters, and their further influence on droplet dynamics may be explored within higher-resolution or multiscale modeling frameworks.

We controlled the resolution by varying the number of grid cells in the radial, axial, and circumferential directions. We tested four grid levels in this study. We simulated the coalescence of droplets with a diameter *D*_d_ = 60 μm, and we compared the liquid–solid contact area under different resolutions. As shown in Figure 2, when the grid count increased from 3.63 million to 4.84 million, the liquid–solid contact area still exhibited a difference of 4.72%, indicating insufficient resolution and limited accuracy. However, when we further increased the grid count from 4.84 million to 8.87 million, the variation in liquid–solid contact area became negligible, and the results converged. Previous studies have reported that when the grid size is approximately 1/25 of the droplet diameter, it is possible to capture the critical details of droplet dynamics adequately.^30^ To better reveal the influence of the wettability gradient on droplet behavior, we used 7.65 million cells for the subsequent simulations, corresponding to a minimum grid size of 0.66 μm. This configuration ensures sufficient computational accuracy while maintaining reasonable efficiency.

**Figure 2:**
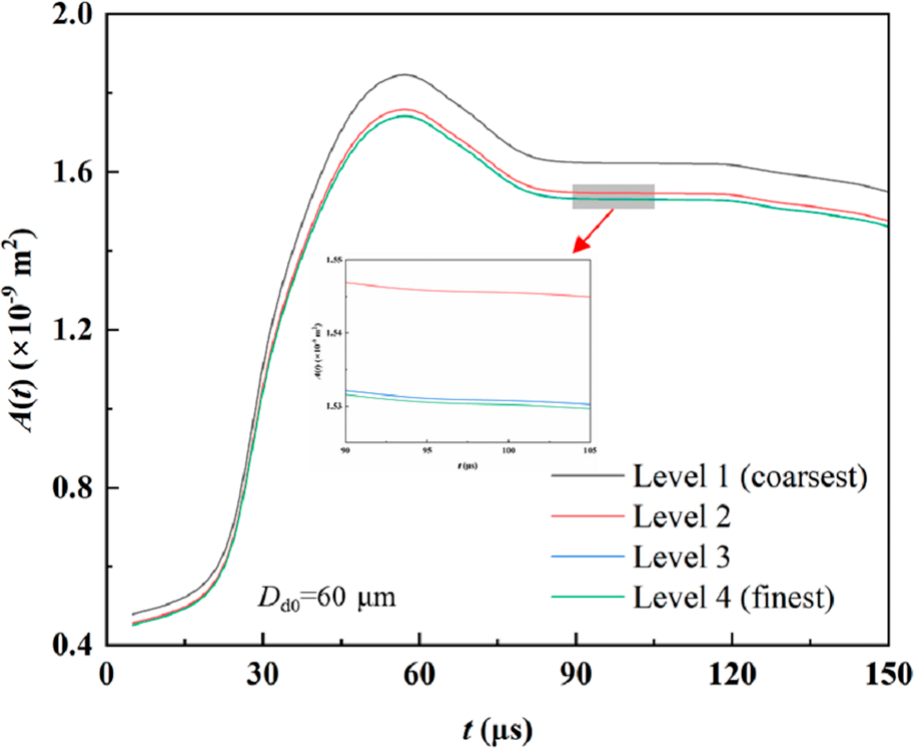
Grid independence verification results.

### 2.3 Boundary Conditions and Solution Methods

This study was based on industrial lubricating oil RCS46, with the droplet density set to 848.5 kg/m^3^, viscosity to 0.069 Pa·s, and surface tension to 0.031 N/m.^31^ The density of compressed air was taken as 3.5 kg/m^3^ with a dynamic viscosity of 2 × 10^–5^ Pa·s, consistent with the actual operating conditions of oil-gas separators.^32^ To investigate viscous effects, the droplet viscosity was further varied within the range of 0.011–0.093 Pa·s, which corresponds to the typical viscosity range of lubricating oils and their oil-mist droplets commonly encountered in industrial coalescence filtration applications. The lower bound of approximately 0.011 Pa·s is representative of relatively light lubricating oils or the same oils operating at elevated temperatures, whereas the upper bound of about 0.093 Pa·s corresponds to higher-viscosity oils or typical low-temperature operating conditions. Numerical simulations were carried out using ANSYS Fluent, with a no-slip boundary condition applied on the fiber surface and pressure outlet conditions on all other boundaries. The wettability behavior was modeled using an improved dynamic contact angle approach, implemented through a user-defined function (UDF).

We calculated the gradient and surface pressure using the node-based Green-Gauss method and the PRESTO! interpolation scheme, respectively. Diffusion terms were discretized with a central difference scheme, while we treated convection terms with a second-order upwind scheme. To improve the efficiency of unsteady simulations, the momentum equations were solved using the non-iterative time advancement (NITA) method, which significantly reduces computational time compared with traditional iterative algorithms.^33^ We based droplet interface tracking on geometric reconstruction, and we employed the piecewise linear interface construction (PLIC) method to represent the interface shape. For numerical stability, we kept the Courant number in the volume fraction equation below 0.1, and we applied an adaptive time step.

### 2.4 Model Validation

The reliability of the numerical method mainly depends on the dynamic contact angle model. Droplet impact on a flat surface was simulated and compared with Dong’s experimental data.^34^ Figure 3 presents the evolution of the droplet height and spreading diameter during the impact. Both the Kistler model and the improved model used in this study agree well with the experiments in the initial spreading stage. However, in the receding stage, the Kistler model neglects the stagnation effect at maximum spreading, leading to an overestimation of the receding speed and reducing the accuracy. In contrast, the improved model shows better consistency with the experimental data throughout both spreading and receding.

**Figure 3:**
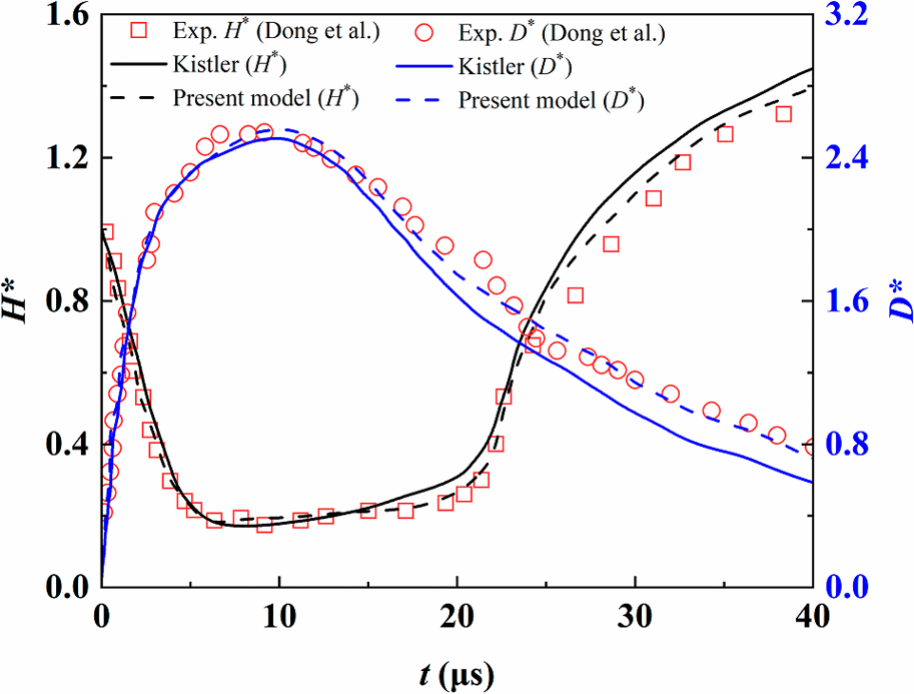
Comparative validation of dynamic contact angle models.

We further compared the simulation results with the experimental data of Liu et al.,^35^ focusing on the process of droplet impact on a single fiber, as shown in Figure 4. The comparison was conducted under identical geometric parameters and operating conditions. The simulated liquid film spreading length agrees well with the experimental measurements. Overall, both the validation of the dynamic contact angle model and the quantitative verification of droplet spreading length demonstrate acceptable agreement with experimental results, confirming the reliability of the simulation model.

**Figure 4:**
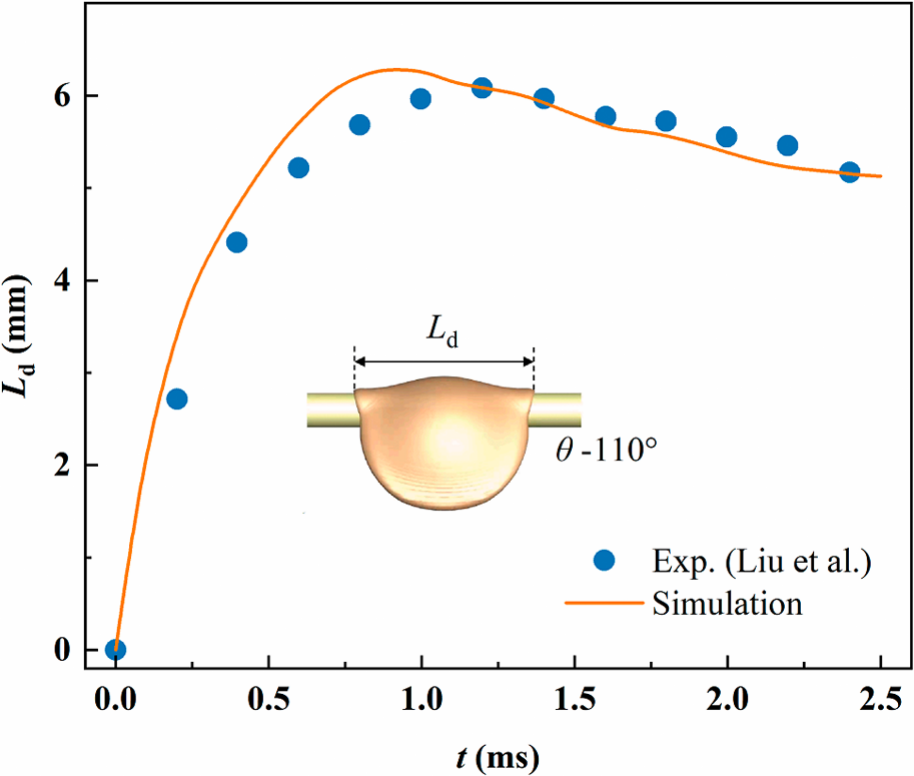
Comparison of simulated and experimental results.

For the transitional regime following maximum droplet spreading, where the contact line velocity is low, the dynamic contact angle model employs a linear empirical approximation. This treatment is introduced to ensure numerical continuity and to represent the overall effect of contact angle hysteresis, with any associated errors primarily confined to the vicinity of the contact line and to short time scales. Model validation demonstrates that, even under this approximation, the simulations are able to reasonably reproduce the experimentally observed droplet coalescence morphology and migration trends, indicating that this treatment does not have a significant impact on the macroscopic migration behavior or the viscous dissipation mechanisms examined in this study.

## 3 Results and Discussion

### 3.1 Morphology of Coalesced Droplets

The coalescence and subsequent directional movement of droplets on the fiber surface are key to understanding the mechanism of wettability gradient-driven motion. During coalescence, the formation of the liquid bridge and the interfacial reconstruction cause rapid morphological evolution of the droplets. To compare the effects of different wettability contrasts and initial configurations on droplet evolution, we analyzed the dynamics of coalescing droplets. At the micrometer droplet scale and the corresponding time scales considered here, droplet coalescence and subsequent migration are primarily governed by the balance between capillary driving forces induced by the wettability gradient and viscous resistance within the droplet. Compared with surface tension effects, gravitational forces are weak at this scale, and the influence of the surrounding gas flow on the initial coalescence and short-time migration can be neglected. Gravitational effects and gas-phase flow may become comparable to capillary and viscous forces only when the droplet size increases to the millimeter scale, when the fiber orientation leads to a significant gravitational component along the migration direction, or when strong background flow and pronounced aerodynamic shear are present.

Figure 5a illustrates the coalescence and subsequent evolution of droplets under a wettability gradient of 150°–90°, considering two distinct initial configurations. A baseline case with an initial droplet diameter of 60 μm and viscosity of 0.069 Pa·s was used for comparison. In both cases, whether the droplets are placed side by side along the fiber or positioned on opposite sides, they first undergo coalescence and then exhibit directional migration driven by the gradient. However, compared with the droplet position at 300 μs, we can observe that the droplet exhibits a slightly faster directional movement when placed along the fiber. In contrast, Figure 5b shows the case of a uniform wettability condition 120°–120°, where droplets from both initial configurations coalesce without displaying directional migration along the fiber, confirming that the wettability gradient is the critical driving factor for droplet motion.

**Figure 5:**
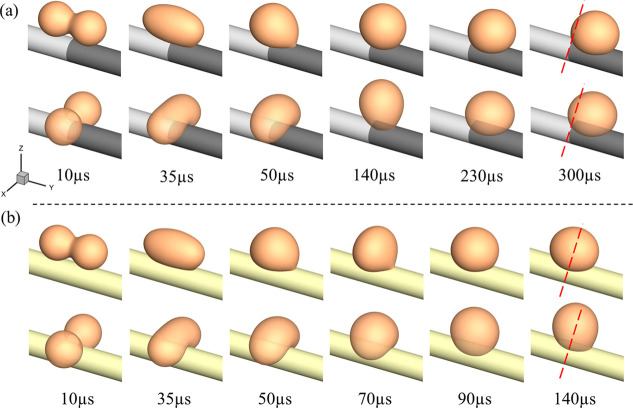
Morphological evolution of coalesced droplets with different initial positions: (a) wettability gradient 150°–90°; (b) uniform wettability 120°–120°.

The cylindrical surface of the fiber introduces anisotropy in the distribution of inertia and surface tension along the circumferential and axial directions of the droplet. Figure 6 shows the temporal evolution of the normalized droplet dimensions in three directions for two initial positions, where *L* denotes the coalescence direction and *H* represents the height perpendicular to the fiber axis. For initial position II, the droplets are located on opposite sides of the fiber, where the geometric confinement is more pronounced and the fiber curvature restricts the expansion of the liquid bridge.^36^ As a result, more significant variations appear in the normal height direction, as evident from the comparison between Figure 6b,e. The influence of the wettability gradient on the dimensional evolution mainly appears in the deformation amplitude. When the overall fiber wettability is higher, the droplet spreading ability increases, leading to larger deformation. Although the overall evolution trends of the droplet morphology are similar for both initial positions, their dynamic behaviors differ due to the combined effects of curvature and geometric confinement.

**Figure 6:**
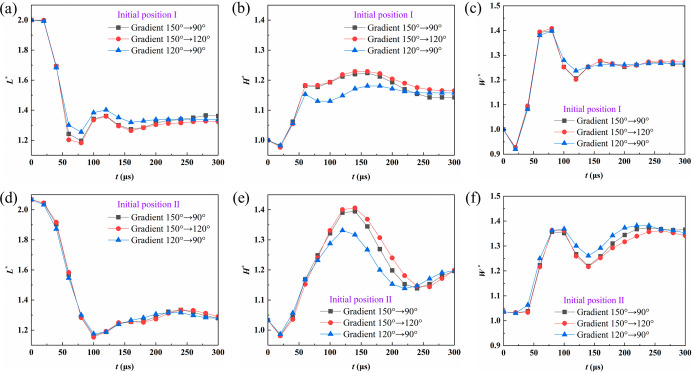
Temporal evolution of the normalized droplet dimensions under different wettability gradients. (a) L*, position I; (b) H*, position I; (c) W*, position I; (d) L*, position II; (e) H*, position II; (f) W*, position II.

Figure 7 demonstrates the temporal variation of the normalized gas–liquid interfacial area *A*_LV_* for the two initial positions. In the early stage of coalescence, the interfacial area decreases rapidly, indicating a fast release of interfacial energy. For initial position I, the area stabilizes at approximately 0.77 at 40–50 μs, corresponding to a reduction of about 23%, suggesting a faster interfacial reconstruction. For initial position II, the decrease is slower. It becomes stable after 80–100 μs with a steady value of about 0.83, showing a slight rebound, which indicates that geometric confinement and fiber curvature delay the release of interfacial energy.

**Figure 7:**
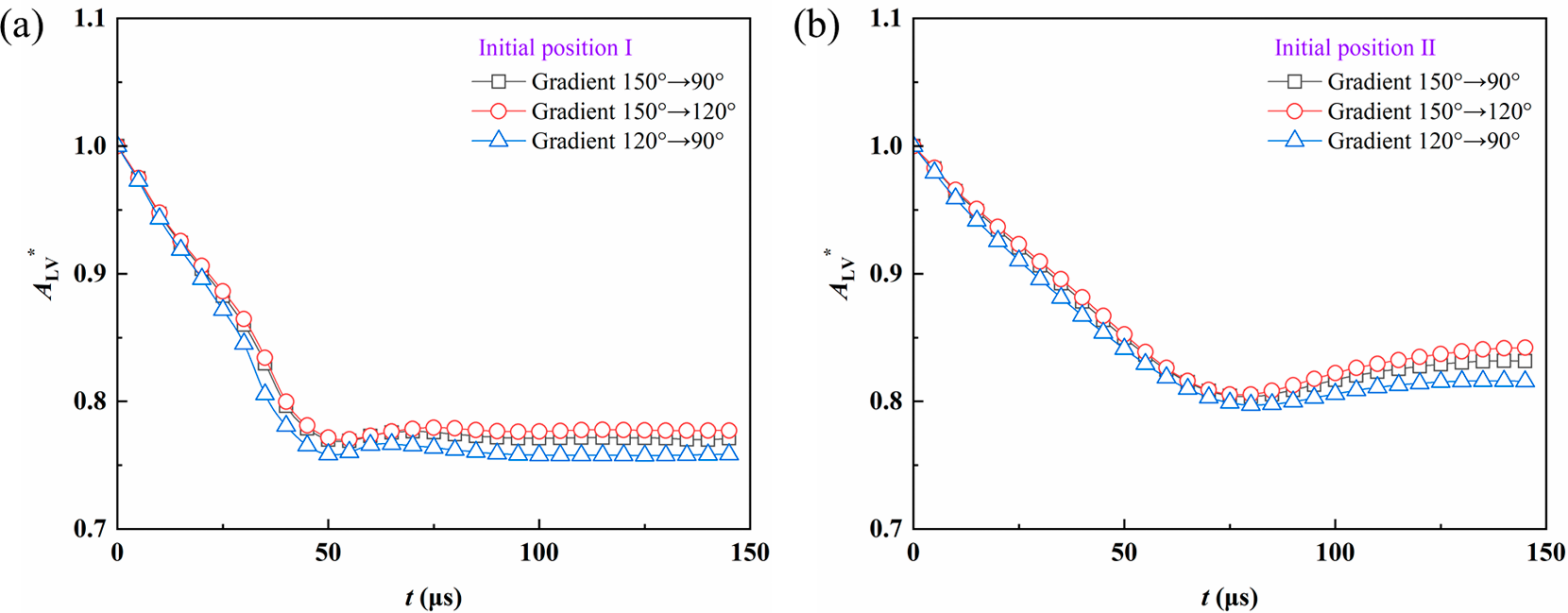
Variation of the normalized gas–liquid interfacial area under different wettability gradients for (a) initial position I and (b) initial position II.

### 3.2 Analysis of Oil Droplet Velocity

The wettability gradient not only affects droplet attachment and coalescence patterns but also determines their directional motion after coalescence. We analyzed the velocity characteristics of droplet coalescence and movement on wettability-gradient fibers. Figure 8 illustrates the influence of wettability gradients and two initial positions on droplet velocity. As illustrated in Figure 8a,b, for initial position I, larger wettability gradients lead to higher droplet velocities along the fiber. Under a 150°–90° gradient, the maximum droplet velocity reaches 0.196 m/s. For the 150°–120° gradient, the peak velocity decreases to 0.155 m/s, and for the 120°–90° gradient, it further drops significantly to 0.054 m/s. In the absence of a gradient (120°–120°), no directional migration occurs. The reason is that the wettability gradient generates an unbalanced driving force along the fiber, which is positively correlated with the surface energy gradient caused by the difference between the advancing and receding contact angles. Therefore, a larger wettability gradient produces a stronger driving force.^21^ For the same contact angle difference, a larger contact angle on the more wettable side results in faster droplet motion. The velocity component in the *z*-direction indicates oscillations of the droplet centroid during coalescence, primarily arising from the alternation between liquid bridge expansion and interfacial retraction.

**Figure 8:**
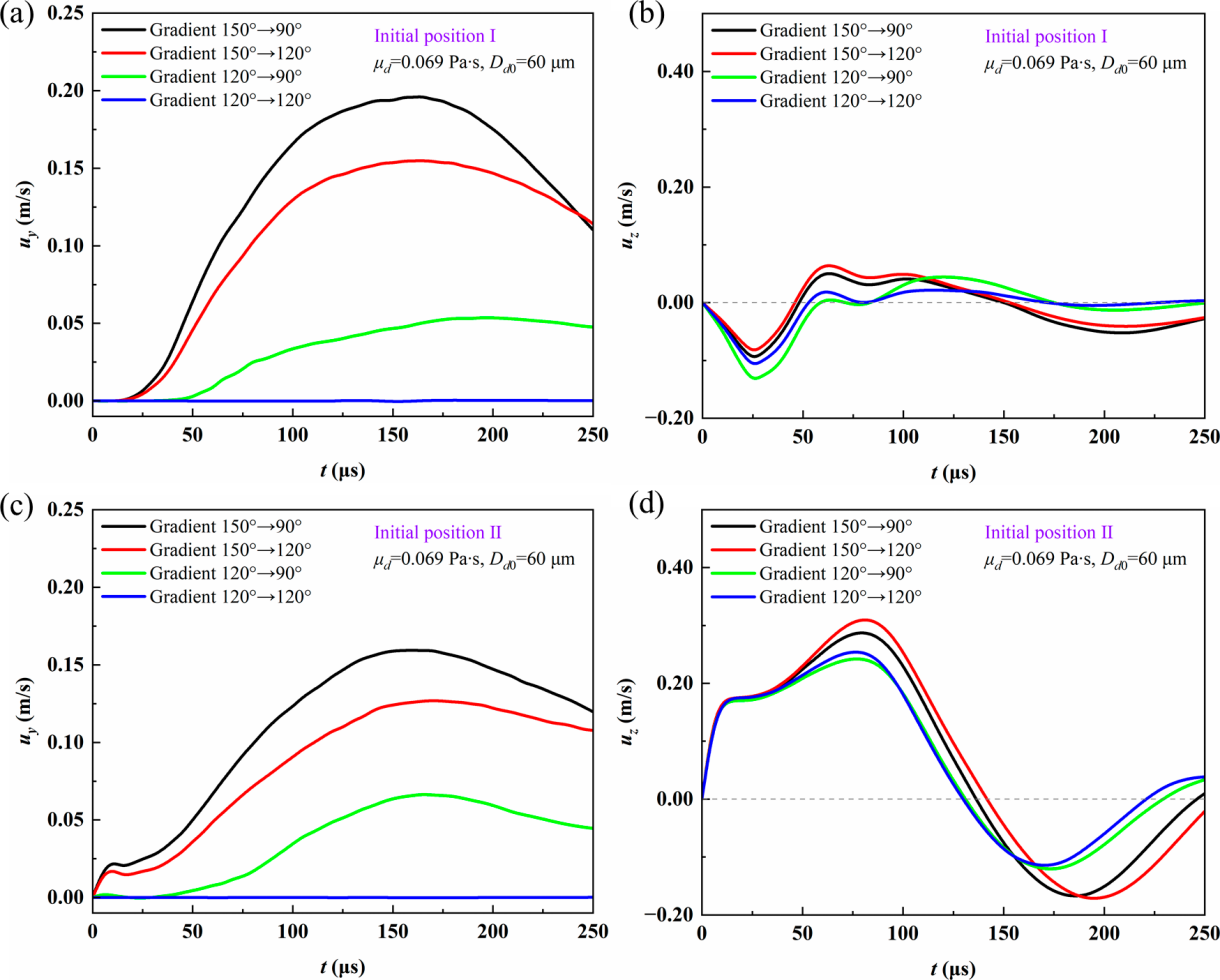
Droplet velocity components under different wettability gradients as functions of time: (a) *u*_*y*_, position I; (b) *u*_*z*_, position I; (c) *u*_*y*_, position II; (d) *u*_*z*_, position II.

As depicted in Figure 8c,d, the velocity trend along the fiber at initial position II is similar to that at position I, but with lower magnitudes. When droplets are distributed on opposite sides of the fiber, the dynamics of liquid bridge expansion and three-phase contact line merging changed, leading to more pronounced oscillations in the *z*-direction. This behavior reflects the combined influence of fiber curvature and opposing droplet configuration on liquid bridge evolution. The initial position governs droplet coalescence and oscillation, whereas the wettability gradient determines the velocity of directional motion.

On fibers featuring a wettability gradient, droplet coalescence and directional transport exhibit characteristic velocity vector signatures. As illustrated in Figure 9, during the initial stage of liquid bridge expansion, the bridge accelerates the surrounding liquid, giving rise to a symmetric flow pattern directed toward the droplet interface center. With continued coalescence, the influence of the wettability gradient transforms the velocity vector distribution from symmetric to asymmetric, causing the internal flow to shift toward the right. Subsequently, the droplet undergoes a net rightward migration, and the velocity field gradually stabilizes, aligning with the fiber surface characterized by the smaller contact angle. The evolution of the droplet velocity field involves a transition from symmetric flow dominated by liquid bridge expansion to asymmetric flow governed by the wettability gradient, ultimately enabling directional motion.

**Figure 9:**
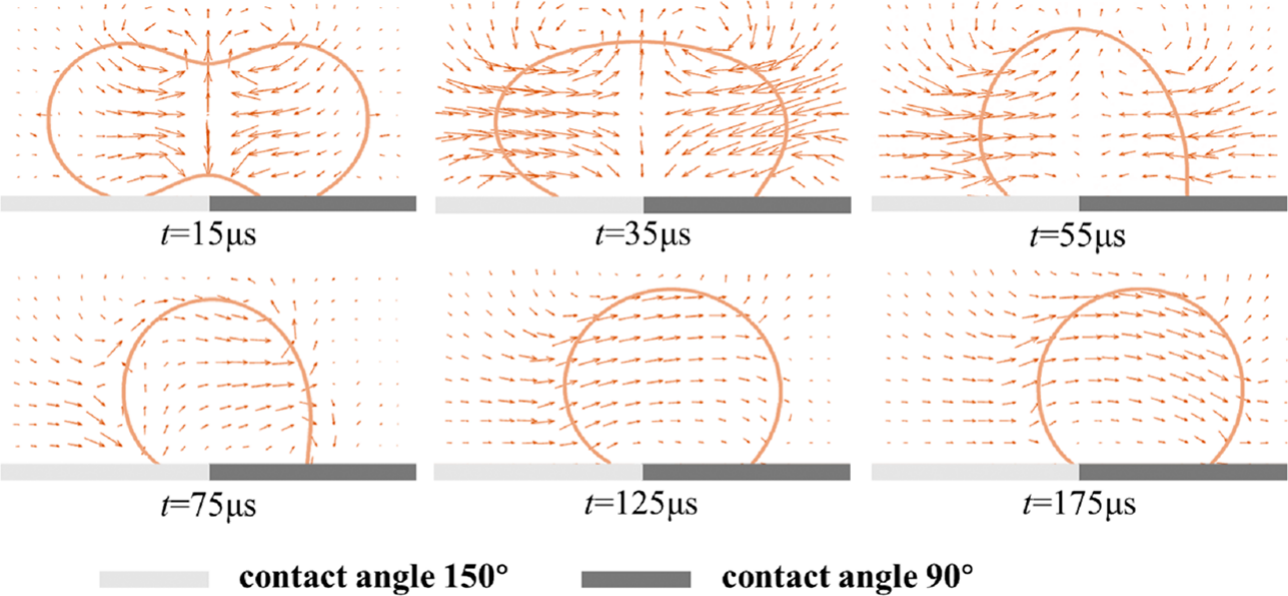
Evolution of velocity vectors during droplet coalescence and directional motion.

To reveal how the wettability gradient affects the internal flow of droplets, it is necessary to examine the characteristics of the velocity gradient. Due to the identical physical mechanism of droplet motion at different initial positions, the following analysis is based on initial position I. Figure 10 shows the spatial distribution of the velocity gradient du_*y*_/d_*y*_ along the *Y* direction at *t* = 15 μs, 55 μs, 75 μs, and 175 μs, used to compare the internal shear flow characteristics of droplets under a 150°–90° wettability gradient and uniform wettability of 120°–120°. As time progresses, the fluctuation amplitude of the curves gradually decreases, and the extrema of velocity gradient weaken accordingly. Under uniform wettability, the curves remain symmetric, and the high velocity gradients are mainly located in the bridge neck region and at the gas–liquid interface.

**Figure 10:**
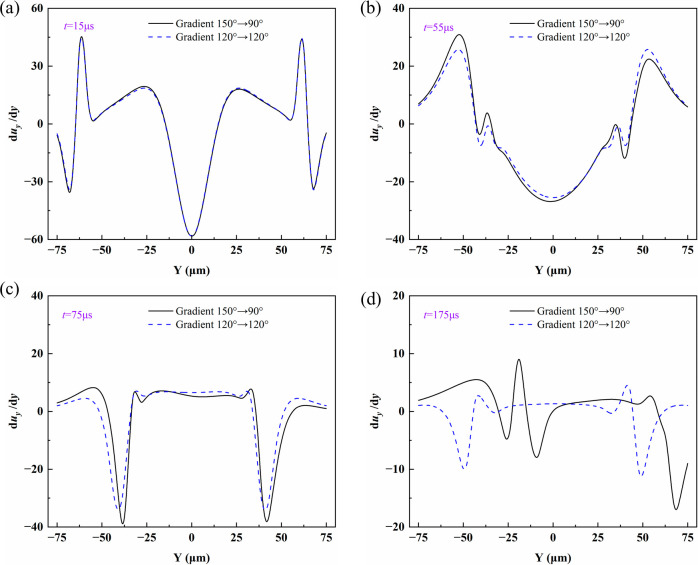
Spatial distribution of the velocity gradient along the *Y* direction with and without a wettability gradient at (a) *t* = 15 μs, (b) *t* = 55 μs, (c) *t* = 75 μs, and (d) *t* = 175 μs.

At *t* = 15 μs, the velocity gradient distributions under both conditions are symmetric with high peak values, showing strong velocity gradient characteristics. At this stage, their trends are nearly identical, and differences in the velocity field are negligible. At *t* = 55 μs, the overall magnitude decreases, and the flow becomes biased due to the driving effect of the wettability gradient, indicating that the gradient direction has begun to influence the local flow. By *t* = 75 μs, the internal flow of the droplet is further restructured. At *t* = 175 μs, the velocity gradient distribution under the wettability gradient exhibits oscillatory decay, while the velocity gradient under uniform wettability nearly approaches zero. The wettability gradient changes the internal velocity gradient distribution of the droplet and breaks the symmetry of the flow field.

Figure 11 shows the time evolution of the energy conversion ratios of droplets on the fiber under two wettability gradients, 150°–90° and 150°–120°, including surface energy (SE), kinetic energy (KE), and dissipation energy (DE). The total energy is given by TE = SE­(*t*) + KE­(*t*) + DE­(*t*).^37^ At the initial moment *t* = 0 μs, the total energy of the droplet consists entirely of surface energy, which is then gradually converted into kinetic energy and dissipation energy. The decay rates of surface energy under both gradients are generally similar, but under the larger wettability gradient of 150°–90°, more surface energy is converted into kinetic energy. At *t* = 175 μs, under the 150°–90° gradient, kinetic energy accounts for 5.29% and dissipation energy for 7.32%. Under the smaller wettability gradient of 150°–120°, kinetic energy accounts for 3.30% while dissipation energy rises to 9.10%. Combined with the earlier analysis of velocity gradients and velocity vectors, a stronger wettability gradient reduces viscous dissipation and increases kinetic energy conversion efficiency, enhancing droplet transport capability.

**Figure 11:**
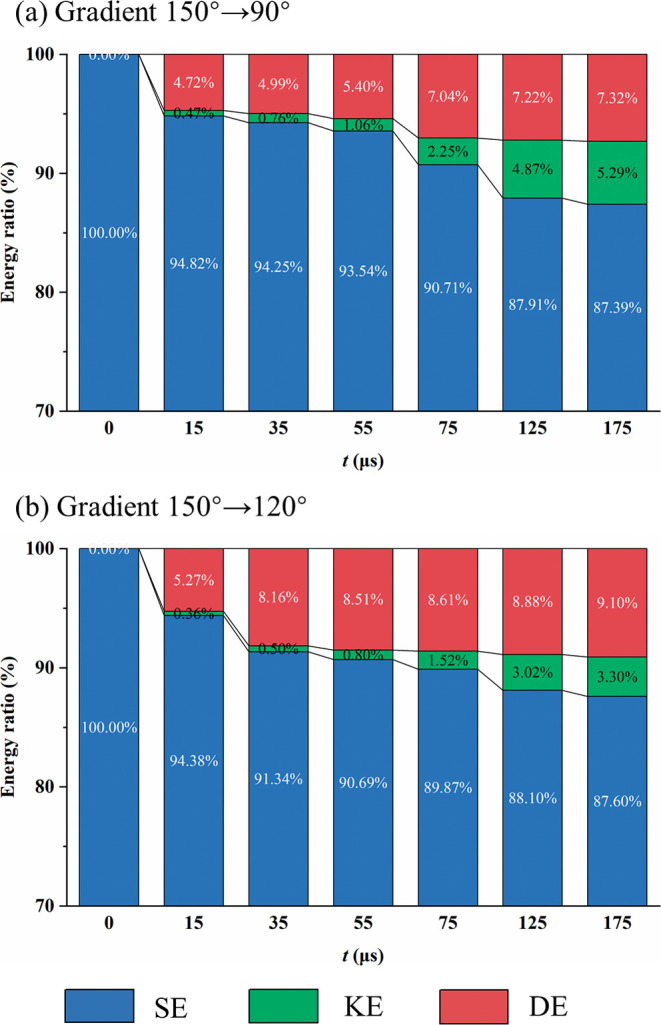
Energy conversion ratios of droplets under different wettability gradients: (a) 150°–90° and (b) 150°–120°. SE, surface energy; KE, kinetic energy; DE, dissipation energy.

During droplet coalescence and migration at the micrometer scale, the system typically operates in a strongly dissipative regime dominated by capillary and viscous effects. As a result, only a limited fraction of the released surface energy is converted into appreciable kinetic energy or resolvable dissipative energy. Consequently, the relatively low numerical proportions of kinetic and dissipative energies reported in this study represent a characteristic feature of such systems rather than an indication of limited energy-transfer efficiency in wettability-gradient-driven processes. Instead, most of the energy is dissipated through viscous mechanisms, which suppress excessive inertial effects and thereby enable stable droplet migration and controlled drainage, an outcome that is advantageous for practical coalescence filtration applications.

### 3.3 Effect of Droplet Viscosity

To analyze the viscous effects in droplet motion driven by the wettability gradient, it is necessary to examine the influence of droplet viscosity on the dynamic characteristics. Viscosity not only determines the droplet’s deformability but also affects its internal flow structure and energy conversion efficiency, serving as a key factor governing droplet deformation, internal flow patterns, and modes of energy dissipation.^38^ As illustrated in Figure 12, the velocities of oil droplets on the fiber differ markedly under various viscosities. After coalescence, the droplet velocity gradually rises from zero, with viscosity exerting a pronounced influence. The maximum velocity of the low viscosity droplet with a viscosity of 0.024 Pa·s is 0.288 m/s, while the high viscosity droplet with a viscosity of 0.093 Pa·s reaches only 0.173 m/s. The velocity curve of the low viscosity droplet fluctuates more strongly, showing oscillations during acceleration, whereas the high viscosity droplet exhibits a smoother increase due to stronger dissipation.

**Figure 12:**
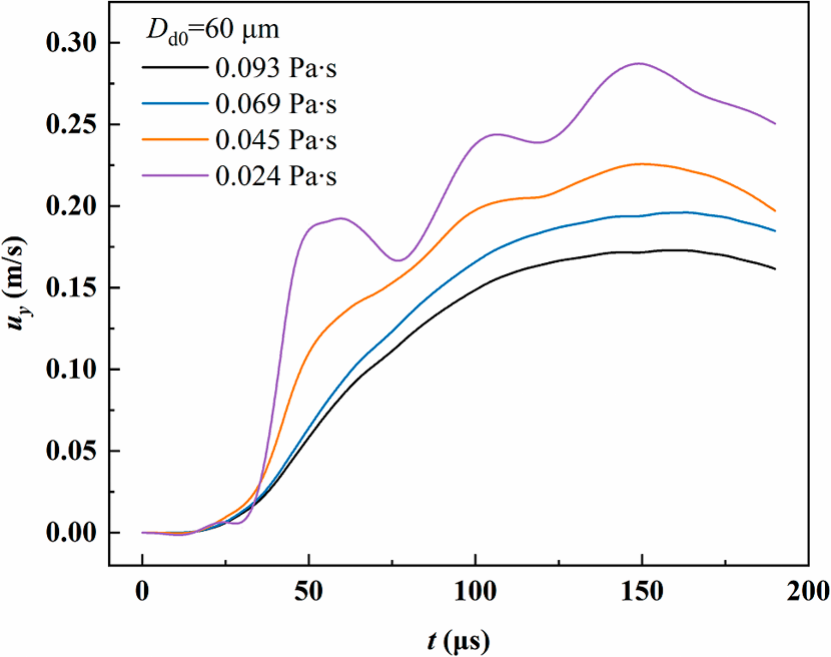
Variation of the droplet velocity along the fiber during coalescence and migration under different viscosities.

The total viscous dissipation rate (TVDR) quantifies the rate of kinetic energy loss due to internal viscous forces during droplet motion. In studies of droplet migration along wettability gradient surfaces, TVDR provides a measure of the energy dissipated as a result of internal viscous resistance. TVDR is calculated by integrating the local viscous dissipation rate (VDR) over the droplet volume, expressed as^39^

8 $${TVDR=\int }_{V_{l}}\phi dV$$

here, Φ represents the local viscous dissipation rate, which includes terms related to the velocity gradient and the strain rate

9 $$\begin{array}{l} \phi =\mu \;[2{\left(\frac{\partial u}{\partial x}\right)}^{2}+2{\left(\frac{\partial \nu }{\partial y}\right)}^{2}+2{\left(\frac{\partial w}{\partial z}\right)}^{2}+{\left(\frac{\partial u}{\partial y}+\frac{\partial \nu }{\partial x}\right)}^{2}+\\ {\left(\frac{\partial \nu }{\partial z}+\frac{\partial w}{\partial y}\right)}^{2}+{\left(\frac{\partial w}{\partial x}+\frac{\partial u}{\partial z}\right)}^{2}] \end{array}$$

To further understand the internal viscous dissipation of droplets during the coalescence and migration process, we divide the VDR term in eq 9 into two components. The first three terms correspond to the normal strain-related dissipation, denoted as TVDR­(N), while the last three terms represent the shear strain-related dissipation, denoted as TVDR­(S).^40^ Figure 13 presents the temporal evolution of the total TVDR and its normal and shear components for droplets with different viscosities during coalescence and migration on the fiber surface. As indicated in Figure 13a, TVDR increases rapidly at the early stage of droplet coalescence (*t* < 10 μs) and reaches its peak at approximately 5 μs. Multiple oscillations occur in the range of 20–60 μs, followed by a rapid decay that stabilizes around 90 μs. As the liquid viscosity increases from 0.024 Pa·s to 0.093 Pa·s, the peak value of TVDR gradually rises. At the same time, the oscillation amplitude decreases, indicating that higher viscosity enhances energy dissipation and suppresses interfacial inertial oscillations. Figure 13b illustrates that the normal strain dissipation rate, TVDR­(N), dominates the total viscous dissipation. Its peak position coincides with that of the TVDR, accounting for approximately 63% of the total viscous dissipation at the peak stage, which corresponds to the formation of the liquid bridge and the strong overall deformation of the droplet. In Figure 13c, the TVDR­(S) is significantly smaller and mainly originates from tangential flows near the contact line and along the fiber surface.

**Figure 13:**
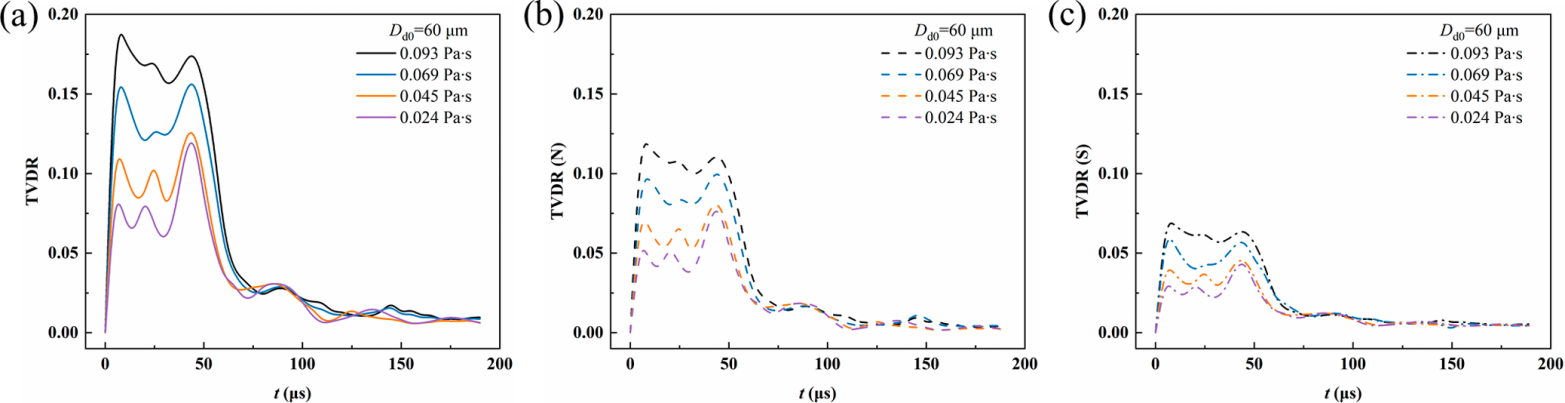
Evolution of TVDR and its components during droplet coalescence and migration on the fiber for different viscosities: (a) total TVDR; (b) TVDR­(N), normal strain-related dissipation; (c) TVDR­(S), shear strain-related dissipation.

Overall, during the early stage of coalescence, the rapid expansion of the liquid bridge leads to pronounced viscous dissipation. Between 20 and 60 μs, capillary-inertial coupling induces oscillations in energy, after which viscous damping becomes dominant and the dissipation rate gradually stabilizes. With increasing viscosity, the internal energy loss becomes larger, and the droplet deformation and oscillation processes become smoother, indicating that viscous resistance plays a key suppressive role in regulating the droplet migration dynamics driven by the wettability gradient.

Figure 14 compares the variation of the velocity gradient along the *Y* direction at viscosities of 0.024 Pa·s and 0.045 Pa·s. At all times, the velocity gradient magnitude of the lower-viscosity fluid is consistently higher than that of the higher-viscosity fluid, indicating that higher viscosity significantly suppresses shear intensity and the rate of velocity variation in the flow field. The low-viscosity fluid maintains a stronger shear gradient response, while the high-viscosity fluid, due to stronger viscous dissipation, reaches a steady velocity gradient earlier. This is consistent with the deformation and internal flow characteristics of viscous droplets.^41^

**Figure 14:**
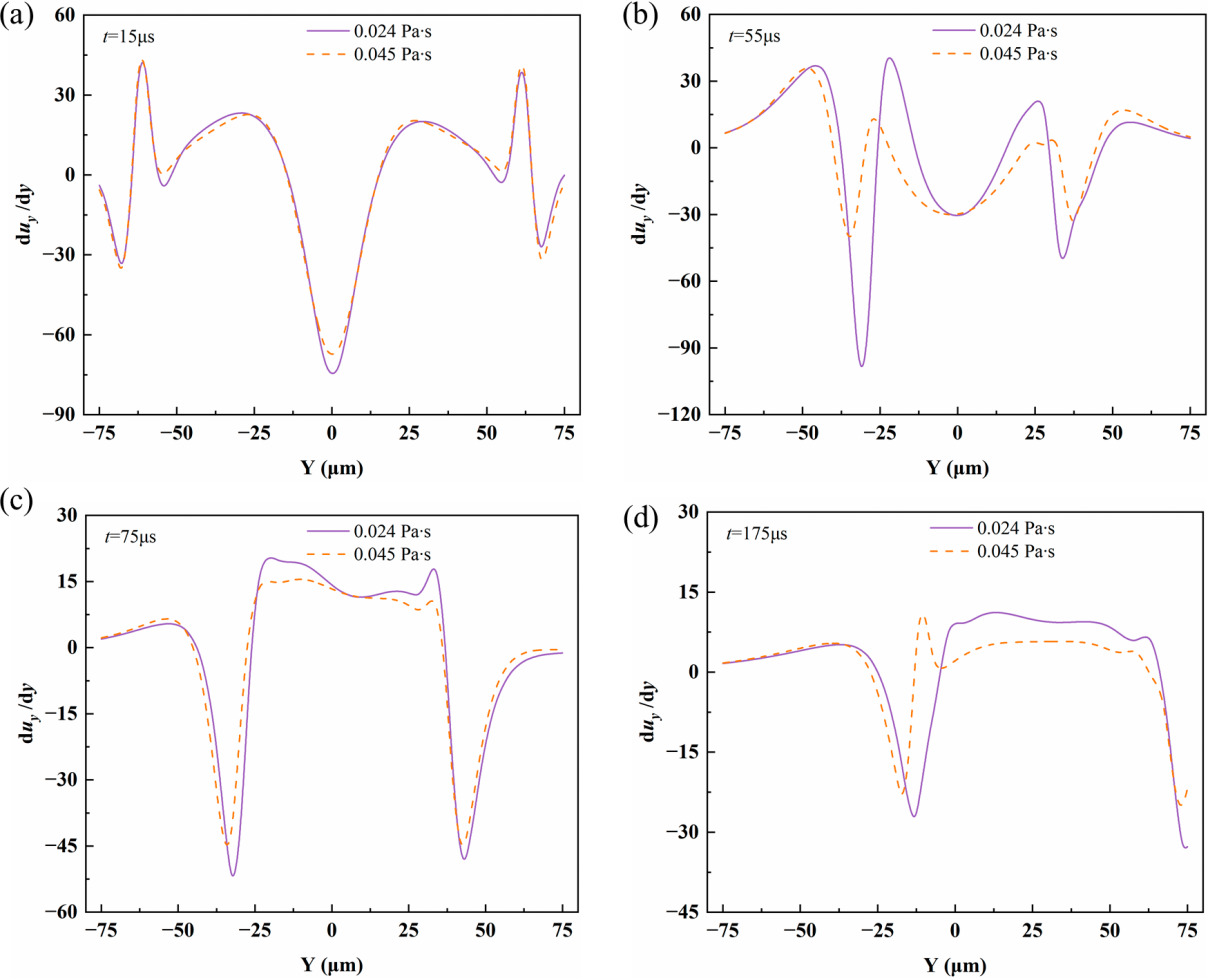
Spatial distribution of the velocity gradient along the *Y* direction for droplets with viscosities of 0.024 and 0.045 Pa·s at (a) *t* = 15 μs, (b) *t* = 55 μs, (c) *t* = 75 μs, and (d) *t* = 175 μs.

By comparing the variation trends of the maximum droplet velocity under different viscosities and wettability gradients, the competing mechanisms between viscous resistance and wettability-driven force, as well as their influence on the droplet migration dynamics, can be clarified. Figure 15 presents the variation of the maximum velocity of oil droplets on the fiber with droplet viscosity under different wettability gradients. As viscosity increases, the maximum droplet velocity decreases, indicating that viscous resistance suppresses the self-propelled motion of the droplets. At the same viscosity, a larger wettability gradient leads to a higher velocity. The velocity under the 150°–90° gradient is consistently greater than that under the 150°–120° gradient. In contrast, the 120°–90° gradient yields the lowest velocity, suggesting that both the contact angle difference and the larger contact angle on one side jointly determine the driving force.

**Figure 15:**
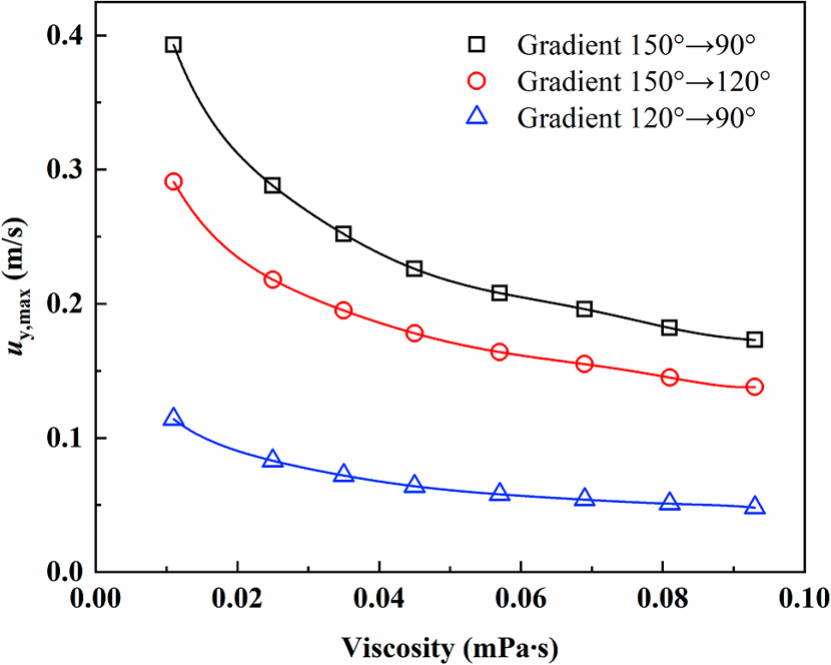
Variation of the maximum velocity of oil droplets on the fiber with droplet viscosity.

The sensitivity of the maximum droplet velocity to viscosity variation is analyzed. We calculated the sensitivity as the relative rate of change of droplet velocity with respect to viscosity. Within the entire viscosity range, the average sensitivity of the maximum velocity to viscosity is approximately 7.5%. In the low viscosity region of 0.011–0.045 Pa·s, the velocity decreases by more than 40%, corresponding to a sensitivity of about 0.13. In contrast, in the high viscosity region of 0.045–0.093 Pa·s, the sensitivity decreases to about 0.045, and the velocity variation becomes more gradual. This behavior indicates that in the low viscosity region, the droplet motion is more sensitive to viscosity changes, characterized by a strong competition between driving and resistive forces. It should be emphasized that the reported average sensitivity represents an overall measure across the entire viscosity range rather than a uniform response. The pronounced difference between the low- and high-viscosity regimes highlights a clear nonlinear dependence of the migration dynamics on viscosity, with the velocity response becoming progressively damped as viscous dissipation dominates.

In contrast, in the high viscosity region, the system gradually transitions to a viscosity-dominated regime. As the wettability gradient increases, the overall droplet velocity rises. At the same time, the sensitivity to viscosity slightly decreases, suggesting that a stronger wettability-driven force not only enhances the migration capability but also slightly weakens the suppressive effect of viscous resistance.

The results provide insights for the design of fibrous coalescence filtration materials. Introducing a wettability gradient along the fiber direction can effectively drive directional droplet migration after coalescence, which helps accelerate liquid drainage and reduce the risk of liquid retention on the fiber surface. In practical filtration materials, such wettability gradients can be realized at the fiber scale through surface chemical modification, control of coating thickness, or functionalization treatments. Droplet viscosity has a significant influence on migration velocity and energy dissipation, indicating that when treating high-viscosity oil mists, stronger wettability control or optimized fiber structures are required to maintain efficient drainage performance. These findings provide a physical basis for the design of high-efficiency coalescence filtration materials based on wettability regulation.

## 4 Conclusions

This study investigates the coalescence and directional migration dynamics of oil droplets on wettability gradient fibers using the VOF method coupled with an improved dynamic contact angle model. The main conclusions are as follows.

(1) Regardless of whether the droplets are placed side by side along the fiber or positioned on opposite sides, they first undergo coalescence and then migrate directionally along the fiber driven by the wettability gradient. The wettability gradient affects the internal velocity gradient distribution of the droplet and breaks the symmetry of the flow field. A larger wettability gradient leads to a higher migration velocity. For the initial configuration I, the maximum velocity reaches 0.196 m/s under the 150°–90° gradient, while configuration II exhibits a similar trend but with a lower overall velocity. Under the same gradient condition, droplets with larger contact angles move faster.
(2) Low viscosity droplets exhibit pronounced oscillations during the acceleration stage, while high viscosity droplets show smoother velocity growth due to enhanced energy dissipation. As the viscosity increases from 0.024 Pa·s to 0.093 Pa·s, the peak value of TVDR rises and the oscillation amplitude decreases, indicating that higher viscosity strengthens energy dissipation and suppresses interfacial inertial oscillations. The TVDR­(N) dominates the total viscous dissipation, with its peak coinciding with that of the TVDR and accounting for about 63% of the total dissipation, corresponding to the liquid bridge formation and strong overall droplet deformation stage.
(3) As viscosity increases, the maximum droplet velocity decreases, indicating that viscous resistance suppresses the self-propelled motion of the droplet. Within the studied viscosity range, the average sensitivity of the maximum velocity to viscosity is about 7.5%. The droplet motion is more sensitive to viscosity in the low viscosity region, while in the high viscosity region the system gradually transitions to a viscosity-dominated regime. A stronger wettability-driven force slightly weakens the suppressive effect of viscous resistance and increases kinetic energy conversion efficiency.

It should be noted that the present study is conducted based on a single-fiber configuration and does not account for collective phenomena that may arise in multifiber networks, such as droplet bridging, channeling, and network saturation. The advantage of this approach lies in its ability to isolate and elucidate the fundamental physical mechanisms governing droplet migration driven by wettability gradients and viscous dissipation under controlled conditions, thereby providing a mechanistic basis for understanding droplet behavior in more complex fibrous structures. Future work will further incorporate multifiber interactions, background gas flow, and network-scale effects.
